# Eco‐friendly biodegradable film based on kombucha mushroom/corn starch/parsley extract: Physicochemical and antioxidant/antibacterial properties

**DOI:** 10.1002/fsn3.4411

**Published:** 2024-08-16

**Authors:** Zahra Shiri, Sajad Pirsa, Jafar Farzi

**Affiliations:** ^1^ Afagh Higher Education Institute of Urmia Urmia Iran; ^2^ Department of Food Science and Technology, Faculty of Agriculture Urmia University Urmia Iran

**Keywords:** antimicrobial properties, biodegradable film, kombucha mushroom, modified corn starch, parsley extract

## Abstract

The main purpose of this study was to produce biodegradable film based on kombucha mushroom (KM), so kombucha mushroom was grown and used to prepare biodegradable film. Glycerol (Gl), corn starch (St), and parsley extract (PE) were used to improve the characteristics of the kombucha mushroom‐based film. The physicochemical, thermal, and antibacterial properties of the films were investigated using different techniques. The obtained results showed that starch significantly increased the tensile strength of the film (3 Mpa) and glycerol improved the flexibility of the film (70%). Starch increased the film's resistance to dissolution in water, and parsley extract and starch improved water vapor permeability. The pure film of kombucha mushroom had good antioxidant (40% ± 2%) and antibacterial properties, and parsley extract significantly increased these properties of the film, so that the prepared film can be considered as an active film. Starch had no significant effect on antioxidant and antibacterial properties. The pure kombucha mushroom film had cracks on the surface, and the addition of starch removed these cracks and made the structure of the film more homogeneous. Electrostatic interactions between kombucha mushroom, glycerol, starch, and parsley extract were confirmed by Fourier transform infrared spectroscopy (FTIR) results. The pure film of kombucha mushroom was a completely amorphous film, which glycerol, parsley extract, and starch improved the crystallinity of the film. Glycerol and parsley extract decreased the thermal resistance of the film, but starch increased this property significantly (about 40°C), so that the kombucha mushroom/glycerol/starch/parsley extract composite film had the highest thermal resistance. In addition to having acceptable mechanical, thermal, and structural properties, the film based on kombucha mushroom can be used as an active film in the packaging of food products sensitive to microbial and oxidative spoilage due to having suitable antioxidant and antimicrobial properties.

## Graphical abstract

As a new source of production of biodegradable polymers and due to the cheap and reproducible source of kombucha mushroom, this material was used to prepare the film. Kombucha mushroom film was composited with glycerol (Gl), corn starch (St), and parsley extract (PE). KM/Gl/St/PE film showed antibacterial/antioxidant activity. KM/Gl/St/PE film showed good mechanical properties and resistance against water. Due to the antioxidant and antibacterial properties of the prepared films, these films can be used as active films in food packaging. Kombucha tea (a), prepared kombucha mushroom (b), and samples of prepared films (c).

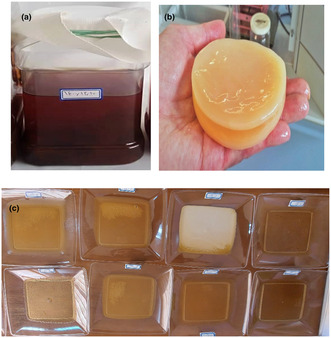

## 1 INTRODUCTION

Nowadays, due to the environmental effects of synthetic and petroleum polymers, the preparation of biodegradable films and coatings for packaging is being considered. The use of biodegradable polymer in the packaging industry, while having the characteristics of normal plastics during their useful life, after a certain period of time, they are converted into natural products such as CO_2_, water, ethane, and biomass by microorganisms. These polymers do not leave heavy elements and toxic substances in the environment. Therefore, they can partially or completely replace synthetic plastics (Almasi et al., 2015; Daei et al., 2022; Petkoska et al., 2021; Pirsa & Mohammadi, 2021). These biodegradable polymers can maintain the quality of foods and increase their stability and durability by limiting unwanted transfers in food products such as the transfer of gases (O_2_ and CO_2_) for fruits and vegetables (Abdolsattari et al., 2020, 2022; Fazeli et al., 2022; Yadav et al., 2023; Yorghanlu et al., 2022).

Kombucha is a fermented and polysaccharide product that is the result of symbiosis between bacteria and yeasts. These yeasts produce fructose and glucose by breaking down sucrose. Kombucha or tea mushroom consists of two parts: the cellulose layer floating on the surface and the fermented liquid. The beneficial effects of this product fermentation on health have increased the consumption of this product (Antolak et al., 2021; Villarreal‐Soto et al., 2018). Kombucha mushroom has the ability to produce biodegradable film due to its polysaccharide structure (Antolak et al., 2021; Vargas et al., 2021).

Natural polymers such as starch have attracted a lot of attention due to their high biodegradability, cheap availability, safe consumption, and easy use. Starch is one of the most abundant and cheapest polymers in nature, which consists of glucose units arranged in two forms of amylose and amylopectin. The functional characteristics of starch are related to the presence of these two macromolecules with high molecular weight and their physical form in the granular structure of starch. The ability to form a starch film is attributed to the amylose content in it. In recent years, several researchers have investigated the use of starch from different sources to produce films and food coatings and determined the functional and mechanical characteristics of the resulting films. In the meantime, films made from corn starch have shown good performance. Today, with the development of food products, modified corn starches with new functional characteristics have been marketed (Colussi et al., 2017; Hernandez‐Perez et al., 2021).

Glycerol is a simple compound (sugar alcohol). Because it is colorless, odorless, and solvent, it has been widely used in the formulation of biodegradable polymers. The strength of glycerol is central and fundamental in all triglyceride compounds. Glycerin is used as a plasticizer and lubricant in film and biopolymer production industries, as well as in paper production. It is also used in the textile industry to soften fabric (Ben et al., 2022; Tarique et al., 2021).

Parsley is a very good source of vitamin A, vitamin C, and vitamin K. In addition, parsley is a good food source of iron and folic acid. The first important component in parsley is volatile oils. These oils are also called essential oils or plant essences (Ajmera et al., 2019). Among the important compounds of this group, we can mention myristicin, limonene, alpha togen, and eugenol. Also, parsley leaf extracts have antimicrobial effects against different bacterial strains (El‐Borady et al., 2020). Parsley extract, due to its antioxidant and antimicrobial properties, when used in biodegradable films, can create biodegradable active films.

In this research, as a new source of production biodegradable polymers and due to the cheap and reproducible source of kombucha mushroom, which is a polysaccharide material and has the ability to form a biodegradable film, this material was used to prepare the film. To improve structural strength, starch was added to it and glycerol was added to increase flexibility. Due to the extraordinary properties of parsley essential oil, this substance was also used to increase the antioxidant and antimicrobial properties of the film. Finally, the physicochemical properties of the film were investigated and the results showed that the prepared films have a suitable physical structure and due to the antioxidant and antibacterial properties of the prepared films, these films can be used as active films in food packaging.

## 2 MATERIALS AND METHODS

### 2.1 Chemicals

In this research, kombucha mushroom and homogenous liquid of kombucha mushroom were purchased from Probiotic online store (Tehran, Iran). Parsley extract was obtained from a medicinal plant shop in Urmia (Iran). Corn starch, glycerol, calcium sulfate, diphenylpicrylhydrazyl (DPPH), and other chemical reagents were obtained from Merck (Germany) and Sigma (USA) companies and were used without additional purification and processing.

### 2.2 Preparation of kombucha mushroom

**FIGURE 1:**
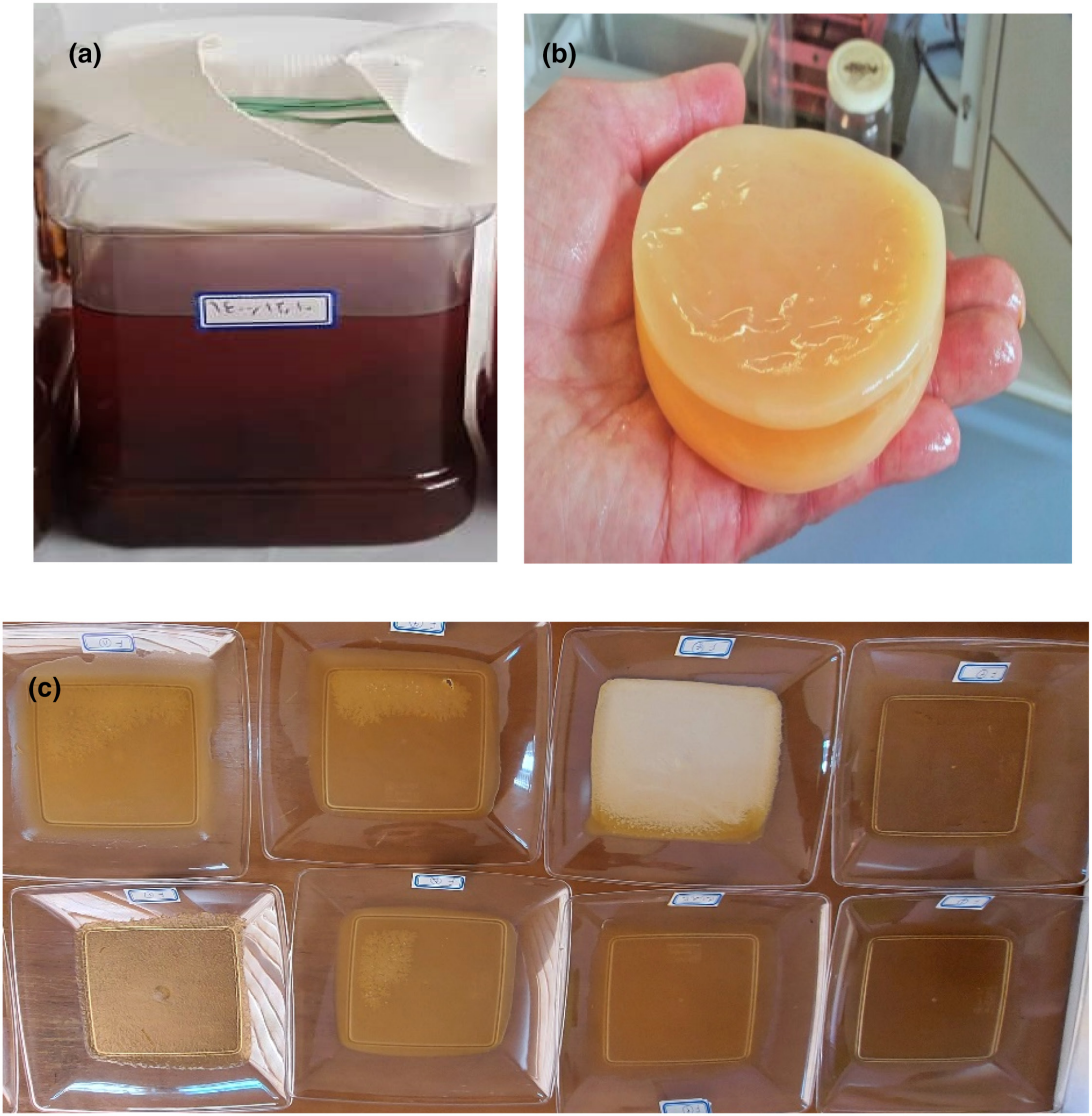
Kombucha tea (a), prepared kombucha mushroom (b), and samples of prepared films (c).

To prepare kombucha mushroom, first, 2 g of dry tea (Iranian Do‐ghazal tea) was boiled in 3 l of city water, and then it was completely filtered and the tea scum was separated. Then, 300 g of sugar was added to the boiled tea solution. Kombucha mushroom (three layers) and starter (apple cider vinegar: 5 mL) were added to the tea solution. The prepared mixture was transferred to special 5‐l containers and the lids of the containers were covered with white cloth and kept for 7 days at ambient temperature and dark conditions to complete the mushroom fermentation process (Figure 1a). The mixture was then passed through a paper filter and the grown kombucha mushroom was isolated (Figure 1b). The separated solids were completely dried in an oven (T 5050 EK Heraeus) at 80°C. The dried mushrooms were completely powdered by the mill and stored in special bags in the refrigerator for later use. Also, the remaining liquid from the mixture was kept in closed containers in the refrigerator until the next use.

### 2.3 Kombucha mushroom film preparation

To prepare the biodegradable film of kombucha mushroom, 5 g of kombucha mushroom powder was mixed in 100 mL of homogeneous liquid prepared from the previous step. The produced homogeneous mixture was placed on a heater–stirrer (RS3001) with a speed of 600 m/s at a temperature of 60°C for 1 h. Then different concentrations of parsley extract, corn starch and glycerol (according to central composite deign; Table 1a) were added to the mixture and the mixture was stirred again for another 30 min at 60°C. Then, the prepared mixture was poured into plastic containers measuring 2 × 15 × 15 cm and kept at room temperature for 72 h (Figure 1c). After this period, the fully dried films were separated from the molds and kept in special zipped bags in the dark until the next tests. Based on the statistical design, 20 films were provided and tested.

**TABLE 1a:** List of films prepared based on the central composite design.

Film	Glycerol (%)	Starch (%)	Parsley extract (%)
1	0	20	0.1
2	0	0	0.1
3	20	10	0.05
4	20	20	0
5	10	10	0.05
6	10	10	0.05
7	20	0	0.1
8	10	10	0.05
9	0	20	0
10	10	10	0.05
11	10	10	0
12	20	20	0.1
13	10	10	0.1
14	10	0	0.05
15	0	10	0.05
16	10	20	0.05
17	10	10	0.05
18	10	10	0.05
19	20	0	0
20	0	0	0

### 2.4 Physicochemical properties of films

#### 2.4.1 Thickness

The thickness of the prepared films was checked using a micrometer (Insize Digital Outside Micrometer 3108‐25A) with an accuracy of 0.01 mm. The film thickness was measured at five different points of the film and the average thickness was calculated (Pirsa et al., 2023).

#### 2.4.2 Mechanical properties

In order to check the mechanical properties, including elongation at breaking point (EAB) and tensile strength (TS), the film samples were placed in special conditions for 24 h. This special condition included relative humidity RH = 55%. The films were cut in dimensions of 1 × 8 cm and used. The cut films were placed between the two jaws of the machine. The initial distance between the two jaws in the device was 50 mm. The movement speed of the upper jaw compared to the lower jaw was 5 mm/min. After the processing operation, the mechanical characteristics were recorded by a texture measuring device (Zwick/Roell model FR010, Germany) and with the help of a computer (Pirsa et al., 2023).

#### 2.4.3 Solubility in water

To measure the solubility in water, first, the films were cut in dimensions of 4 × 4 cm and dried in an oven at 105°C. Then, the weight of the samples was recorded (*W*_*i*_). Then, the film was placed in the Erlenmeyer flasks with a water volume of 50 mL of distilled water. The Erlenmeyer flasks were placed on a shaker at a speed of 200 rpm and at a temperature of 25°C for 24 h. Finally, the samples were dried in the oven at 105°C and weighed again after being filtered by filter paper (*W*_*f*_). The obtained weight difference was used to obtain the film dissolution percentage as follows (Pirsa & Mohammadi, 2021):

(1) $$\text{Solubility}\;\left(\%\right)=\frac{W_{i}-W_{f}}{W_{i}}\times 100$$

In this regard, *W*_*i*_ is the initial weight of the film and *W*_*f*_ is the final weight of the film.

#### 2.4.4 Moisture content

For this test, films were prepared in the desired dimensions (3 × 3 cm). The prepared films were kept for 24 h in a desiccator (containing silica gel), at a relative humidity of 55% and at a temperature of 25°C, and then the weight of the film (*W*_*i*_) was measured. Then, the film was placed inside the oven at 100°C for 24 h and its final weight was measured (*W*_*f*_). The following equation was used to calculate the moisture content of the film (Pirsa et al., 2023).

(2) $$\text{Moisture content}\;\left(\%\right)=\frac{W_{i}-W_{f}}{W_{i}}\times 100$$

In this regard, *W*_*i*_ is the initial weight of the film and *W*_*f*_ is the final weight of the film.

#### 2.4.5 Water vapor permeability (WVP)

To perform this test (using the ASTM E96/E96M‐14 standard method), glass vials with a height of 4 cm were used. The average diameter of the vials was 2 cm. In order to set the relative humidity of the vials at zero, calcium sulfate was placed inside the vials. Next, the entrance door of the vials containing calcium sulfate was covered with the desired films and completely sealed with melted wax. To place the prepared vials in 97% humidity, the sealed vials were placed in a desiccator at a temperature of 25°C. A saturated solution of potassium sulfate was placed inside the desiccator to ensure 97% humidity inside the desiccator. Then, the weight of the vials was measured every 24 h. For 7 days and every 24 h, the vials were weighed and the weights were recorded. The linear curve of the changes in the weight of the vials with respect to time was drawn. The linear equation between the weight of the vial and the calculated time and the slope of the line and the regression coefficient was obtained. Water vapor transmission rate (WVTR) was calculated by dividing the slope of the line of each vial by the total surface of the film. The permeability to water vapor was calculated through the following relationship (Briassoulis & Giannoulis, 2018):

(3) $$\mathrm{WVP}=\frac{\text{WVTR}.X}{P\left(R_{1}-R_{2}\right)}$$

In this regard, *X* is the thickness of the film (meters), *P* is the vapor pressure of pure water at 25°C, *R*_1_ is the relative humidity in the desiccator (RH = 97%), and *R*_2_ is the relative humidity inside the vial (RH = 0%).

#### 2.4.6 Antioxidant properties

DPPH radical quenching method was used for this test. For this purpose, 25 mg of each film was dissolved in 4 mL of distilled water for 2 min to prepare the film extract. 0.2 mL of 1 mM methanolic DPPH solution was added to 2.8 mL of film extract solution and mixed completely (with a vortex of 2000 rpm for 2 min). The obtained solution was kept in a dark place for 1 h. A spectrophotometer (Model T60 UV, USA) was used to measure the absorption of solutions. The absorption of the solutions was done at a wavelength of 517 nm. The following relationship was used to calculate the antioxidant percentage (Pirsa & Mohammadi, 2021):

(4) $$\text{Antioxidant activity}\;\left(\%\right)=\frac{A_{b}-A_{s}}{A_{b}}$$

In this regard, *A*_*b*_ is the absorption rate of the control sample (molar DPPH1 methanolic solutions) and *A*_*s*_ is the absorption rate of the sample.

#### 2.4.7 Antibacterial property

The agar diffusion method was used to determine the antibacterial property of the films against *Staphylococcus aureus* and *Escherichia coli* bacteria. At first, films were cut into disks. The diameter of the disks was 15 mm. The cut films were placed inside plates on Mueller–Hinton agar plates containing 10^7^ CFU/m of *S. aureus* ATCC6538 and 10^7^ CFU/m of *E. coli* ATCC13706. Plates containing Mueller–Hinton agar plates, mentioned bacteria, and film were incubated at 37°C for 24 h. Finally, the radius of the halos of nongrowth of bacteria around the films was measured. The halos of lack of growth were measured in millimeters (Osés et al., 2016).

#### 2.4.8 Scanning electron microscopy (SEM)

A scanning electron microscope (ZEISS, SIGMA, Germany) was used to examine the surface morphology and characteristics of the polymer particles in the films. To perform this test, the film was covered with a thin layer of gold, and the morphology test was performed. The accelerator voltage of the device was set to 20 kV. The sample was placed in the special position of the machine and the surface of the sample was photographed with different magnifications.

#### 2.4.9 Fourier transform infrared spectroscopy (FTIR)

The chemical and structural characteristics of the films were investigated by FTIR test using an IR device (Tensor 27, Bruker, Germany). For this purpose, the films were dried and powdered with a special mill and mixed with KBr at a ratio of 1–20. Using a special press machine, the prepared powder was turned into a thin film and placed in the special place of the machine sample. The spectrum of the samples was recorded in the range of 400–4000 cm^−1^. The resolution of the device was 4 cm^−1^.

#### 2.4.10 X‐ray diffraction (XRD) spectroscopy

For this test, the film was placed in the tube of the sample in the X‐ray diffractometer (Kristalloflex D500, Siemens, Germany) and the primary rays were irradiated to the sample and the reflected rays were recorded in the range of angle 2*θ* = 0–80°. Data and spectra were recorded at ambient temperature. Cu Kα was used as the radiation source and the radiation wavelength was 0.154 nm. X‐rays at 40 kV and 40 mA were used in this device.

#### 2.4.11 Thermal gravimetric analysis (TGA)

Thermal gravimetric analysis (TGA) and differential thermal analysis (DTA) tests were used to check the thermal resistance of the films. Thermal analyzer with specifications (Linseis – L81A1750, Germany) was used for this test. For this test, film samples (10 mg) were heated in aluminum cups in the temperature range of 30°C–600°C. The samples were heated under nitrogen atmosphere at 50 cm^3^/min and the heating rate was 10°C/min. TGA and DTA curves were recorded by the device.

### 2.5 Statistical analysis

In this study, to investigate the effect of variable factors, glycerol, starch, and parsley extract on the physicochemical properties of kombucha mushroom films (thickness and mechanical properties, solubility, moisture content, and permeability to water vapor), the central composite statistical design was used. (Table 1a). A completely randomized design (Table 1b) was used to investigate the effect of glycerol, starch, and parsley extract on the antioxidant, antimicrobial, morphology, crystalline properties, and thermal resistance of the films. Design Expert‐10 software was used to design experiments, analyze the obtained answers, and check mathematical models and relationships between variables and answers. Minitab‐16 software was used to compare means in a randomized design (Duncan's test). A 95% probability level was used to check the mathematical models and compare the averages.

**TABLE 1b:** The list of films prepared based on a completely random design.

Film	Glycerol (%)	Starch (%)	Parsley extract (%)
KM	0	0	0
KM/Gl	20	0	0
KM/St	0	20	0
KM/PE	0	0	0.1
KM/Gl/St/PE	20	20	0.1

## 3 RESULTS AND DISCUSSION

### 3.1 Thickness, TS, and EAB

**FIGURE 2:**
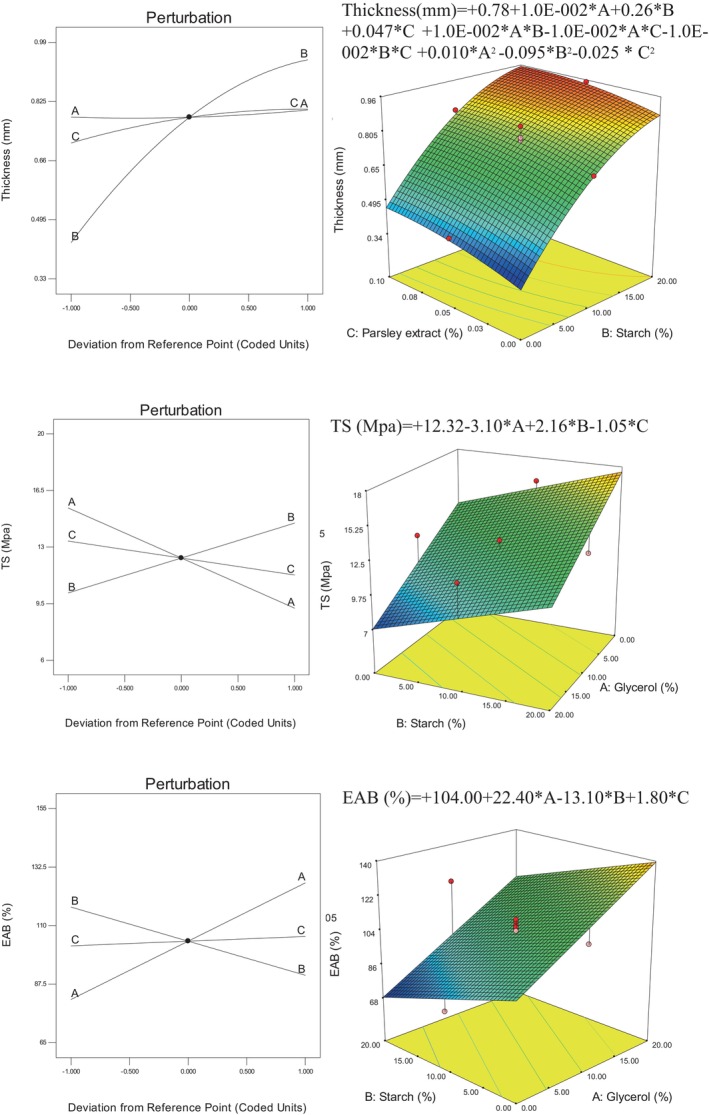
Mathematical models and perturbation and three‐dimensional curves of the effect of glycerol, starch, and parsley extract on the thickness, TS, and EAB of kombucha mushroom composite films.

The thickness and mechanical properties of the films indicate the practical use of biodegradable films in the packaging of various materials, especially food. The more thickness and mechanical and physical resistance the prepared film, the higher its industrial applicability. Figure 2 shows the mathematical models and perturbation and three‐dimensional curves of the three factors of glycerol, starch, and parsley extract on the thickness, TS, and EAB of kombucha mushroom composite films. According to the obtained results, glycerol did not have a significant effect on the thickness of the films, which was probably due to the fact that glycerol filled the empty spaces of the films and did not have a significant effect on the thickness. While starch and parsley extract significantly increased the thickness of the film, the effect of starch in increasing the thickness of the film was greater than that of parsley extract. Considering that the amount of added starch was significant (10% and 20% of dry matter); therefore, an increase in thickness can be expected in the presence of starch, because it has greatly increased the amount of dry matter of the film, which has a direct relationship between the amount of dry matter and the thickness of the film.

In a similar study, Fakhouri et al. (2013) reported that the addition of starch to gelatin film increases the thickness of the film. They reported that with the increase of starch, the dry matter of the film increases and the thickness increases, which confirms the results of the present research.

Examining the tensile strength results showed that only starch increased the tensile strength of the film and glycerol and parsley extract decreased the tensile strength. It can be said that starch has been able to improve the cohesion of polymer chains and increase the tensile strength of the film by creating transverse connections and electrostatic forces, but glycerol as a plasticizer has the opposite effect by weakening the transverse connections and has reduced the tensile strength. Regarding the extract, it can be said that due to the almost hydrophobic properties of the extracts, these materials also do not strengthen the polymer chains, and in many cases, they reduce the strength of transverse connections, which leads to a decrease in tensile strength.

According to the results of EAB, it was expected that due to the plasticizing role of glycerol in biodegradable polymers, adding glycerol to the kombucha mushroom film would increase the stretchability of the film, which is exactly what happened. As mentioned above, parsley extract has also led to an increase in the stretchability of the films by reducing the bonding strength of the polymer chains. Although starch has increased the tensile strength by increasing the links of polymer chains, it has decreased the elasticity of the film.

Although the use of kombucha mushroom to prepare biodegradable films has not been used so far, many studies have investigated the effect of additives on improving the mechanical properties of polysaccharide films. For example, Kit (2023) prepared the biodegradable film of bovine gelatin and found the effect of furfuryl isocyanate and anthocyanin on its mechanical properties. The effect of furfuryl group and concentration of grape anthocyanins (1% and 2% w/v) on the mechanical, structural, thermal, and morphological properties of the films was investigated. The results of his research showed that the mechanical and thermal properties of gelatin films increased in the presence of furfuryl isocyanate and anthocyanin, which is relatively consistent with the results of the current research (Kit, 2023).

### 3.2 Solubility, moisture content, and WVP

**FIGURE 3:**
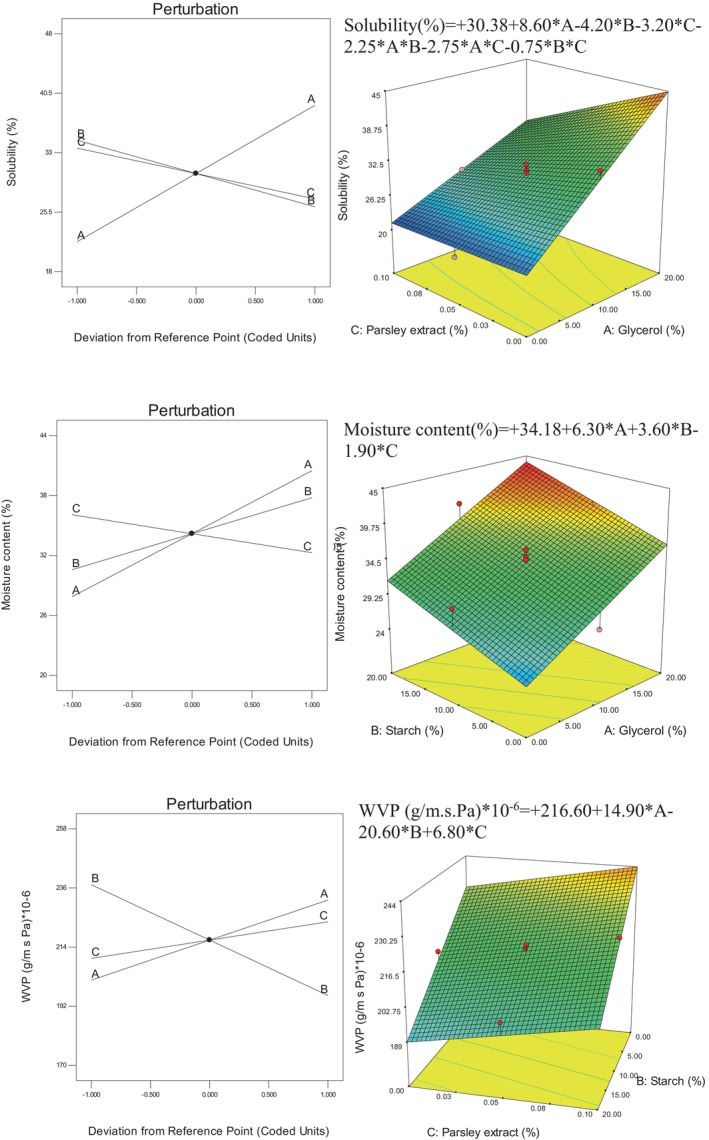
Mathematical models and perturbation and three‐dimensional curves of the effect of glycerol, starch, and parsley extract on the solubility, moisture content, and WVP of kombucha mushroom composite films.

Resistance to water and water vapor is one of the most important drawbacks of biodegradable polymers with the origin of biological materials. Therefore, as much as the water resistance of these biodegradable films can be increased, their industrial usability will increase more and more. The water resistance of biodegradable polymers is of great importance, especially when they are used in the packaging of moisture‐sensitive food or when the packaging is placed in humid environments. Figure 3 shows mathematical models and perturbation and three‐dimensional curves of the glycerol, starch, and parsley extract on solubility, moisture content, and WVP of kombucha mushroom composite films.

By examining the results of solubility in water, it was found that glycerol has increased the solubility in water. Glycerol with its alcoholic structure and having OH groups is easily soluble in water and thus increases its solubility in water. Although glycerol has an adverse effect on the solubility of films, since it increases the flexibility of the film, it is considered an effective substance in the production and preparation of biodegradable films. Starch and parsley extract have also reduced the solubility percentage of the film. Due to the fact that starch has strong electrostatic interactions with kombucha mushroom components, and as it was observed in the examination of the mechanical resistance characteristics, it increases the structural cohesion of the film; therefore, it also increases the film's resistance to water. Parsley extract has also reduced the percentage of film dissolution in water due to its waterproof nature.

Examining the moisture percentage of the films showed that glycerol and starch increased the moisture percentage of the film and parsley extract decreased the moisture percentage. Due to the presence of OH groups, both starch and glycerol can bond with the H of water molecules and trap them in the film structure, which leads to an increase in the moisture content of the film. But the extract has an antiwater nature and probably by filling the spaces between the polymers, it does not allow water molecules to be trapped and lowers the moisture content.

Water vapor permeability in films is a phenomenon that depends on both the hydrophilicity/hydrophobicity of the composite components and the surface and depth gaps of the film. According to the results of water vapor permeability, glycerol and parsley extract have increased water vapor permeability. As mentioned earlier, glycerol weakens the cohesion of polymer chains, provides the necessary space for the passage of water molecules, and increases the permeability to water vapor, but starch does not allow water vapor molecules to penetrate with the cohesion and strength of transverse bonds between the components of the composite and reduces the permeability to water vapor.

Wicakso et al. (2023) investigated the effect of adding chitosan in making an edible film in terms of aqueous properties and determining the best concentration of glycerol. The composition of raw materials was 5 g of corn starch and chitosan composition (0.5%, 1%, and 1.5%). Glycerol concentration was different in different percentages (4%, 5%, and 6%). Their results showed that changing the concentration of glycerol and chitosan strongly affects the mechanical and water properties of the starch film, which shows relative agreement with the results of the current research (Wicakso et al., 2023).

### 3.3 Antioxidant and antimicrobial properties

Antioxidant and antimicrobial properties are two very important properties in films and polymers that place these materials in the category of bioactive materials. Films that have both antioxidant properties and antimicrobial properties, if used as packages for food packaging, can protect the contents of the package from microbial spoilage and oxidative spoilage and increase its storage life or storage time. Increasing the shelf life of food products can prevent wastage of energy and, in addition to preserving energy resources, also prevent the production of pollutants and food waste.

**FIGURE 4:**
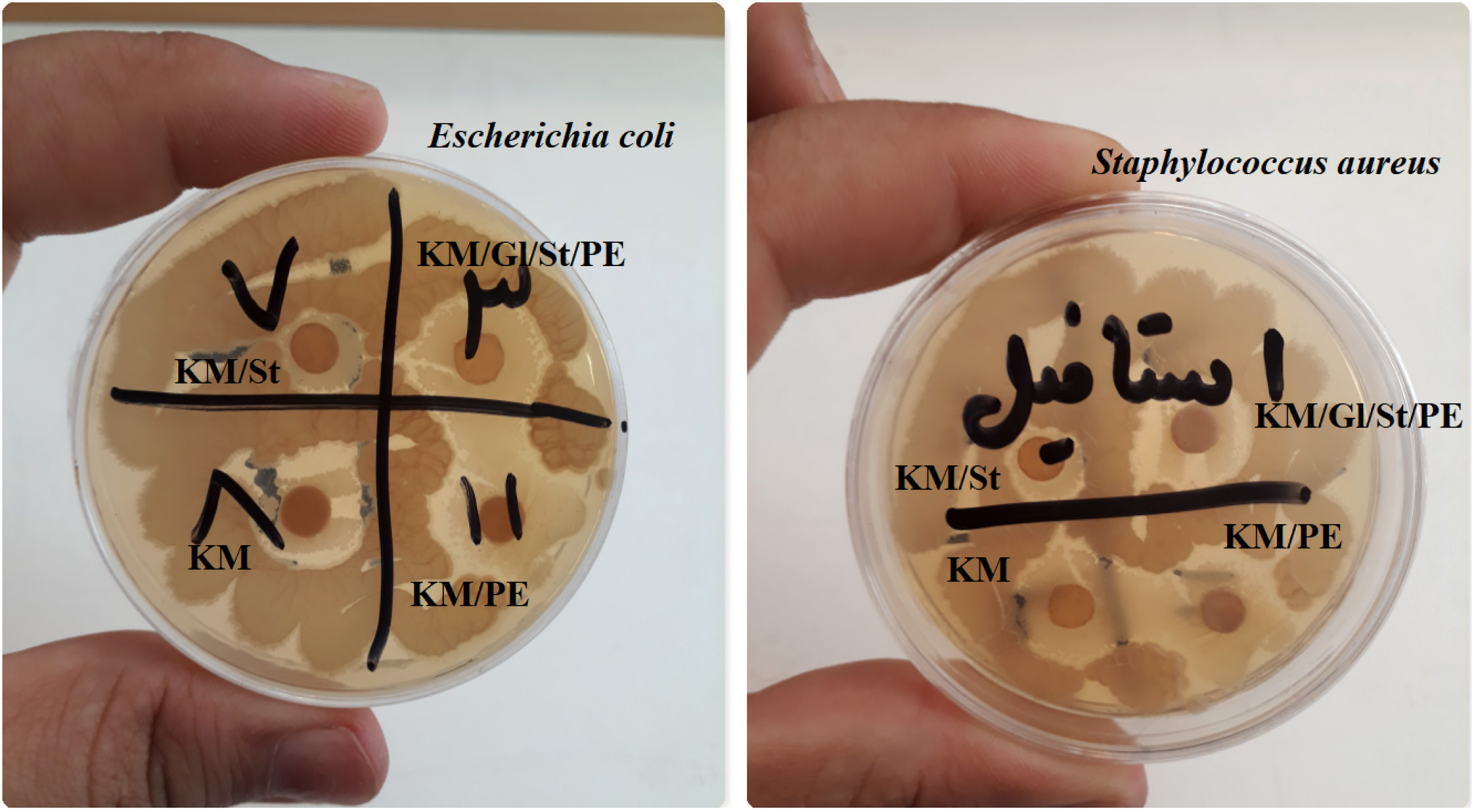
Nongrowth halos of Gram‐positive and Gram‐negative bacteria in the presence of kombucha mushroom film and its various composites.

Table 2 shows the antibacterial properties of kombucha mushroom film and its various composites on Gram‐positive (*S. aureus*) and Gram‐negative (*E. coli*) bacteria. In this Table, the antioxidant properties of the mentioned films are also reported. Also, Figure 4 shows the nongrowth halos of Gram‐positive and Gram‐negative bacteria against different composite films. According to the results of the antioxidant properties, the pure kombucha mushroom film shows good antioxidant properties. Considering that there are various polyphenolic and antioxidant compounds in kombucha mushroom, it was not far from expected that the film of pure kombucha mushroom has good antioxidant properties (Nyiew et al., 2022).

**TABLE 2:** Antioxidant and antimicrobial properties of kombucha mushroom composite film and its composites.

Film	Antibacterial activity: Inhibition zone diameter (mm)		Antioxidant activity (%)
	*Escherichia coli* (G−)	*Staphylococcus aureus* (G+)	
KM	7 ± 0.3^b^	8 ± 0.4^b^	40 ± 2^a^
KM/St	5 ± 0.4^a^	6 ± 0.3^a^	41 ± 3^a^
KM/PE	11 ± 0.4^c^	12 ± 0.2^c^	76 ± 2^b^
KM/Gl/St/PE	10 ± 0.3^c^	11 ± 0.5^c^	77 ± 3^b^

*Note*: Different letters in each column indicate the significance of the difference in means.

It has been reported that this mushroom contains B group vitamins and folic acid, as well as osmic acid, which has antioxidant properties. By comparing different films, it was found that starch did not have a significant effect on the antioxidant property, but parsley extract greatly enhances the antioxidant property. Plant extracts have many polyphenolic compounds that have good antioxidant properties. Moftah et al. (2024) have investigated and confirmed the bioactive compounds of parsley extract and its antioxidant properties. According to their report, parsley leaf extract and its seed oil have significant antioxidant properties, which are in good agreement with the results of the current research (Moftah et al., 2024).

According to the results of Table 2 and Figure 4 related to the nongrowth halos of Gram‐positive and Gram‐negative bacteria in the presence of different films, it was determined that the pure kombucha mushroom film had antimicrobial properties against both bacteria. By comparing the antibacterial results of different films, it was found that the addition of starch to the film has somewhat weakened the antimicrobial properties, which is probably due to the fact that the starch in the film structure involves antimicrobial compounds and prevents their effect on bacteria. But parsley extract has strengthened the antimicrobial effect to some extent. The antibacterial properties of plant extracts have been investigated and confirmed in various studies (Punoševac et al., 2021).

The most antibacterial property was related to the kombucha mushroom film containing parsley extract. Also, by examining and comparing the effects of different composite films on Gram‐positive and Gram‐negative bacteria, it was found that the films had more and better effects on Gram‐positive bacteria (*S. aureus*) than Gram‐negative bacteria (*E. coli*). Considering that Gram‐negative bacteria have a more complex wall structure than Gram‐positive bacteria, they are more resistant to antibacterial agents, and therefore, in general, antibacterial films have a greater effect against Gram‐positive bacteria, and the aura of nongrowth is larger. Misic et al. (2020) have extracted the phytochemical compounds of parsley by supercritical fluid extraction method and studied its antibacterial properties. The research results of this group confirm the results of the current research to a large extent (Misic et al., 2020).

### 3.4 SEM imaging and FT‐IR spectroscopy

**FIGURE 5:**
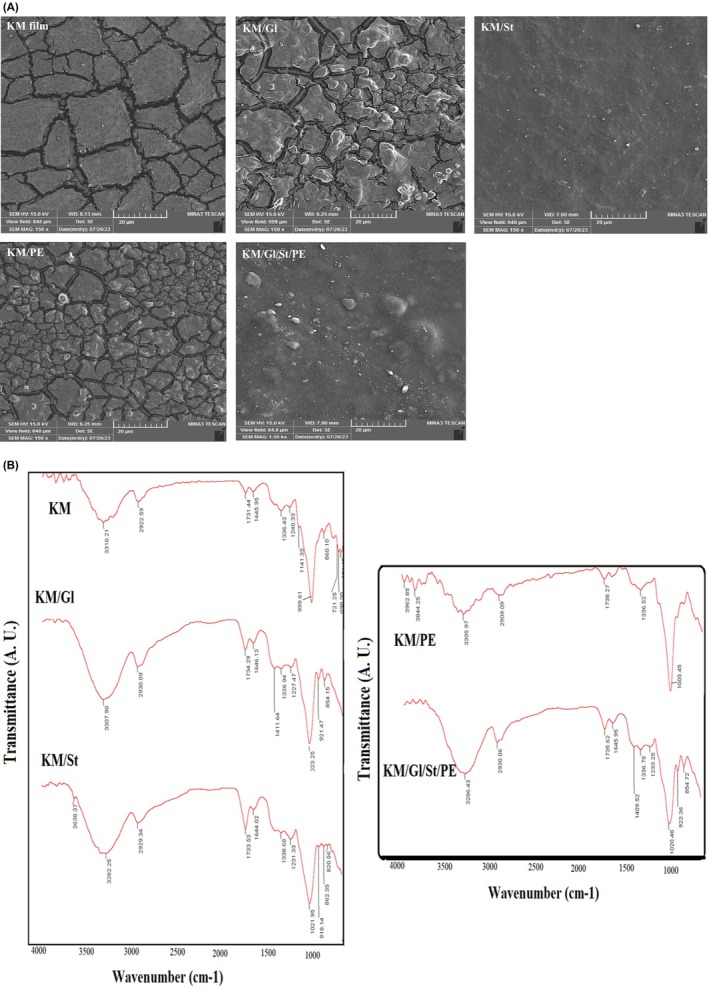
SEM images (a) and FTIR spectra (b) of kombucha mushroom film and its various composites.

Figure 5 shows SEM images and FTIR spectra of kombucha mushroom film and its various composites. According to the SEM images, the kombucha film has cracks on the surface that are clearly visible. Considering that kombucha is a polysaccharide layer containing various types of yeasts and bacteria; therefore, the film prepared from this mushroom is also a kind of polysaccharide film and considering that no polymer binders are used in the pure film of kombucha mushroom, the existence of surface cracks on the film surface was somewhat expected.

In kombucha mushroom films containing plasticizer glycerol and parsley extract, surface cracks are still observed on the surface of the film, and especially in kombucha film containing parsley extract, the extract particles are placed between the polysaccharide chains of the kombucha film and increase the surface cracks. In kombucha film containing starch, cracks and surface cracks are much less and the surface of the film is more uniform. It can be said that in films containing starch, starch acts as a connector between polymer chains and composite components and has reduced the surface and deep cracks of the film. It should be mentioned that the reduction of surface cracks and fissures in biodegradable films leads to the reduction of permeability to water vapor, which is a desirable process in the process of preparing films and polymers.

According to FTIR spectra, the formation of kombucha mushroom film and its composites and electrostatic interactions between composite components and no chemical change in the structure of composite components are confirmed. Thus, in the FTIR spectrum of kombucha mushroom film, the peak at 3310 cm^−1^ is related to the stretching vibrations of O—H and N—H groups, which, according to the polysaccharide structure of kombucha mushroom, confirms the possibility of these functional groups in the main structure of the film. In this spectrum, peak 2922 shows C—H and —CH_2_— stretching vibrations, which are abundant in the structure of polysaccharides, proteins, and other hydrocarbons. Peak of 1731 also shows the stretching vibrations of C=O groups, which due to the complex structure of kombucha mushroom and the presence of various compounds in its structure, the presence of C=O groups is completely scientific and expected. Peak 1645 also shows the stretching vibrations of C=O groups related to R—COR. Peaks 1336, 1240, 1141, 999, and 860 are, respectively, related to the stretching vibrations of N—O, C—O, O—R groups, and C—H out‐of‐plane vibrations (Ma et al., 2022; Pinto et al., 2023). In total, the appeared peaks confirm the chemical structure of kombucha mushroom to some extent. By comparing the spectrum of the pure kombucha mushroom film with its different composites, it can be seen that all the peaks related to different functional groups in the structure of the kombucha mushroom in the composites have shifted to different wave numbers, and these changes in wave numbers indicate electrostatic interactions between the components of composites. Also, the absence of new and clear peaks in the spectrum of composite films indicates the absence of chemical reactions or structural degradation in the main structure of kombucha mushroom and composite components. An interesting point that can be seen in the spectrum of kombucha film containing parsley essential oil is that the intensity of the peak in the 3300 region, which is related to the vibrations of O—H and N—H groups, has decreased to a large extent compared to other films, which is probably due to the reason that in the presence of parsley extract, the moisture content of the films has decreased (this result can also be seen in the discussion of the moisture content of the films), which has reduced the intensity of the corresponding peak.

Gao et al. (2019) have investigated various functional groups of polysaccharide films using FTIR technique, and the results of their reports confirm the results of the current research (Gao et al., 2019).

### 3.5 XRD spectroscopy and thermal analysis

**FIGURE 6:**
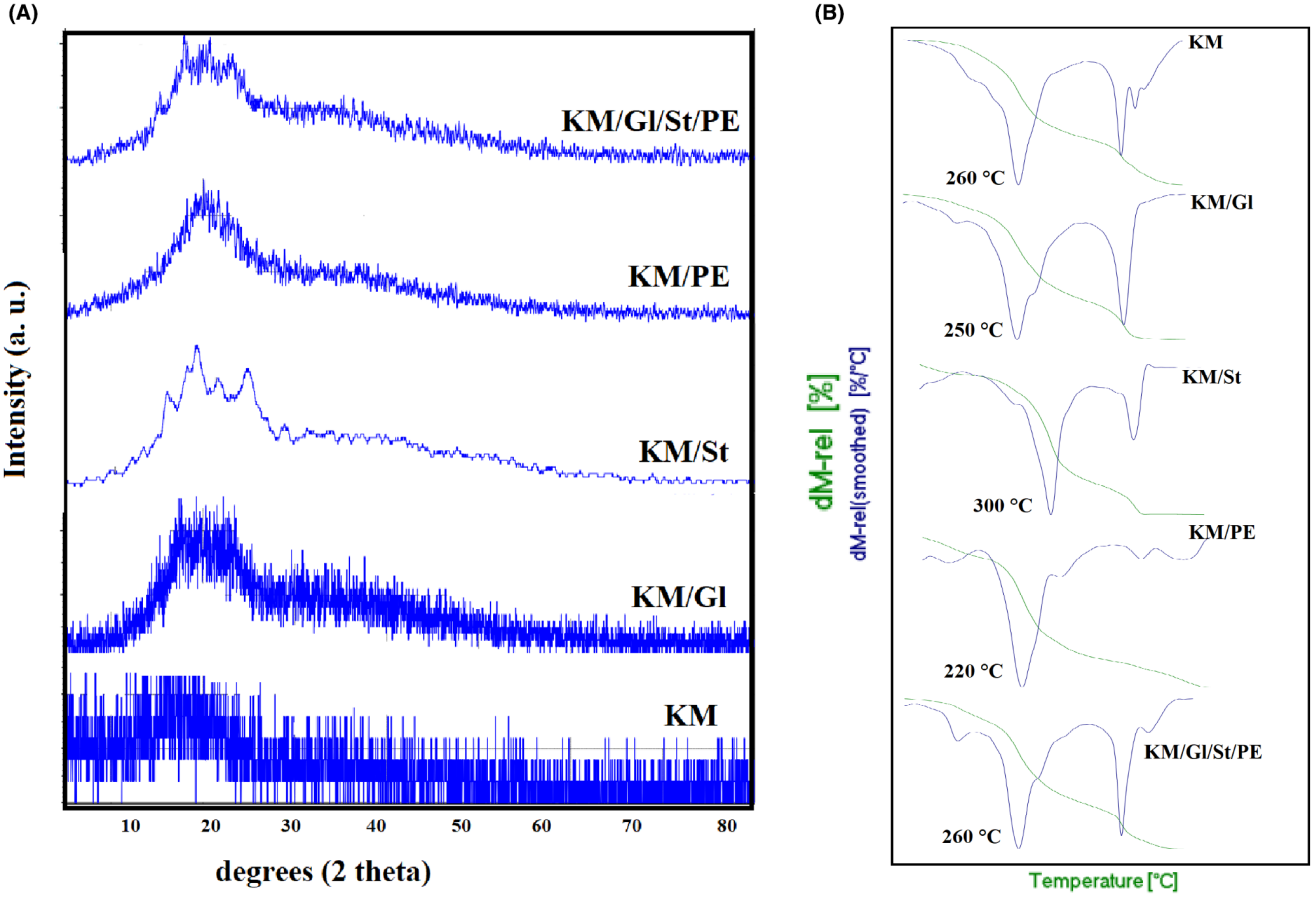
XRD spectrum (a) and TGA/DTA (b) of kombucha mushroom film and its various composites.

Figure 6 shows the XRD spectrum and thermal decomposition curves (TGA and DTA) of kombucha mushroom film and its various composites. According to XRD results, kombucha mushroom film is completely disordered (amorphous) and noncrystalline, and no specific peak is observed in this spectrum. The addition of glycerol, starch, and parsley extract improves the crystalline properties of the film and makes the structure of the film more regular. Among the materials added to the film, corn starch has the greatest effect in increasing the crystalline properties of the film, so that in the kombucha film containing corn starch, several clear and sharp peaks are observed in the range of 2*θ* from 15 to 22°, which indicates the crystalline structure of the composite. The peaks appearing in this area are related to corn starch according to what was reported in previous research (Kibar et al., 2010). It can be said that the starch particles have not only made the structure of the film more coherent, but also improved the structural and crystalline order of the film.

Ali et al. (2019) have investigated the crystal structure of starch‐based biodegradable films. They reported that starch has a crystalline/semicrystalline structure. The peaks reported for the starch film in their research confirm the results of the present research (Ali et al., 2019).

According to the thermal decomposition curves related to kombucha mushroom film and its various composites, thermal decomposition occurs in three stages in all curves. The first stage of thermal decomposition is related to the evaporation of water molecules and glycerol in the film. In the DTA curves, the peak related to this stage in films containing glycerol compared to the pure film of kombucha mushroom (which does not contain glycerol) is clearer and sharper. The second stage of thermal decomposition is related to the breakdown of the overall structure of the film, and the main weight loss also occurs in this stage. The third stage of thermal decomposition, which includes about 10%–15% of the total weight, takes place in the temperature range of 450°C–500°C, and this thermal decomposition is probably related to polysaccharides, proteins, and other organic compounds with high decomposition temperatures that are present in the structure of kombucha mushroom. By comparing the thermal decomposition curves of the kombucha mushroom film with its composites, it can be seen that by examining the second stage of thermal decomposition (which is the main stage of thermal decomposition), it is determined that starch has increased the thermal resistance of the kombucha film up to 50°C, while glycerol and parsley extract have reduced the thermal resistance of the film. As it was clear in the investigation of the morphology of the film and the mechanical properties of the film, starch significantly increased the physical and mechanical resistance of the film, improved the cohesion of the film, and reduced the cracks and fissures of the film. It has led to the improvement of the thermal stability of the film (Lozano‐Navarro et al., 2018; Ruggero et al., 2020). Suriyatem et al. (2018) have investigated the effect of starch in improving the thermal stability of carboxymethyl cellulose film. The results of their research confirm the results of the current research on the thermal stability of kombucha mushroom film in the presence of starch (Suriyatem et al., 2018).

## 4 CONCLUSION

Kombucha mushroom was grown using tea and its powder was used to prepare biodegradable film. Glycerol, corn starch, and parsley extract were used to improve the properties of kombucha mushroom‐based films. Examining the tensile strength results showed that only starch increased the tensile strength of the film and glycerol and parsley extract decreased the tensile strength. By examining the results of solubility in water, it was found that glycerol has increased the solubility in water. Glycerol and starch increased the moisture content of the film and parsley extract decreased the moisture content. According to the results of water vapor permeability, glycerol and parsley extract have increased water vapor permeability. The pure kombucha mushroom film showed good antioxidant properties. Starch did not have a significant effect on the antioxidant property, but parsley extract strongly strengthened the antioxidant property of the film. It was found that the pure film of kombucha mushroom had antimicrobial properties against both bacteria. Parsley extract enhanced the antimicrobial effect to some extent. According to the SEM images, the kombucha film had cracks on the surface. In the kombucha film containing starch, cracks, and surface cracks were much less and the surface of the film became more uniform. The FTIR spectrum confirmed the chemical structure of kombucha mushroom and the electrostatic interactions between the components of the composites to some extent. In addition to making the film structure more coherent, the starch particles also improved the structural and crystalline order of the film. Starch increased the heat resistance of kombucha film up to 50°C. Due to the cheapness of kombucha mushroom and also due to the fact that this mushroom grows and reproduces easily in environmental conditions, it can be used as a suitable and abundant source for the production of biodegradable polymers in the future. It is suggested to investigate the economic aspects of industrial production of packaging films based on kombucha mushroom.

## AUTHOR CONTRIBUTIONS

Sajad Pirsa conceived of the presented idea. Zahra Shiri developed the theory and performed the computations. Sajad Pirsa verified the analytical methods. Jafar Farzi discussed the results and contributed to the final manuscript. Zahra Shiri carried out the experiment. Sajad Pirsa wrote the manuscript and revised it.

## FUNDING INFORMATION

The author(s) received no financial support for the research, authorship, and/or publication of this article.

## CONFLICT OF INTEREST STATEMENT

There is no conflict of interest among authors.

## Data Availability

Research data are not shared.

## References

[fsn34411-bib-0001] Abdolsattari, P. , Pirsa, S. , Peighambardoust, S. J. , Fasihnia, S. H. , & Peighambardoust, S. H. (2020). Investigating microbial properties of traditional Iranian white cheese packed in active LDPE films incorporating metallic and organoclay nanoparticles. Chemical Review and Letters, 3(4), 168–174.

[fsn34411-bib-0002] Abdolsattari, P. , Rezazadeh‐Bari, M. , & Pirsa, S. (2022). Smart film based on polylactic acid, modified with polyaniline/ZnO/CuO: Investigation of physicochemical properties and its use of intelligent packaging of orange juice. Food and Bioprocess Technology, 15(12), 2803–2825.

[fsn34411-bib-0003] Ajmera, P. , Kalani, S. , & Sharma, L. (2019). Parsley‐benefits & side effects on health. International Journal of Physiology, Nutrition and Physical Education, 4(1), 1236–1242.

[fsn34411-bib-0004] Ali, A. , Chen, Y. , Liu, H. , Yu, L. , Baloch, Z. , Khalid, S. , Zhu, J. , & Chen, L. (2019). Starch‐based antimicrobial films functionalized by pomegranate peel. International Journal of Biological Macromolecules, 129, 1120–1126. 30218726 10.1016/j.ijbiomac.2018.09.068

[fsn34411-bib-0005] Almasi, H. , Ghanbarzadeh, B. , Dehghannia, J. , Pirsa, S. , & Zandi, M. (2015). Heterogeneous modification of softwoods cellulose nanofibers with oleic acid: Effect of reaction time and oleic acid concentration. Fibers and Polymers, 16, 1715–1722.

[fsn34411-bib-0006] Antolak, H. , Piechota, D. , & Kucharska, A. (2021). Kombucha tea—A double power of bioactive compounds from tea and symbiotic culture of bacteria and yeasts (SCOBY). Antioxidants, 10(10), 1541. 34679676 10.3390/antiox10101541 PMC8532973

[fsn34411-bib-0008] Ben, Z. Y. , Samsudin, H. , & Yhaya, M. F. (2022). Glycerol: Its properties, polymer synthesis, and applications in starch based films. European Polymer Journal, 175, 111377.

[fsn34411-bib-0009] Briassoulis, D. , & Giannoulis, A. (2018). Evaluation of the functionality of bio‐based plastic mulching films. Polymer Testing, 67, 99–109.

[fsn34411-bib-0010] Colussi, R. , Pinto, V. Z. , El Halal, S. L. M. , Biduski, B. , Prietto, L. , Castilhos, D. D. , da Rosa Zavareze, E. , & Dias, A. R. G. (2017). Acetylated rice starches films with different levels of amylose: Mechanical, water vapor barrier, thermal, and biodegradability properties. Food Chemistry, 221, 1614–1620. 27979137 10.1016/j.foodchem.2016.10.129

[fsn34411-bib-0011] Daei, S. , Mohtarami, F. , & Pirsa, S. (2022). A biodegradable film based on carrageenan gum/*Plantago psyllium* mucilage/red beet extract: Physicochemical properties, biodegradability and water absorption kinetic. Polymer Bulletin, 79(12), 11317–11338.

[fsn34411-bib-0012] El‐Borady, O. M. , Ayat, M. S. , Shabrawy, M. A. , & Millet, P. (2020). Green synthesis of gold nanoparticles using parsley leaves extract and their applications as an alternative catalytic, antioxidant, anticancer, and antibacterial agents. Advanced Powder Technology, 31(10), 4390–4400.

[fsn34411-bib-0013] Fakhouri, F. M. , Costa, D. , Yamashita, F. , Martelli, S. M. , Jesus, R. C. , Alganer, K. , Collares‐Queiroz, F. P. , & Innocentini‐Mei, L. H. (2013). Comparative study of processing methods for starch/gelatin films. Carbohydrate Polymers, 95(2), 681–689. 23648030 10.1016/j.carbpol.2013.03.027

[fsn34411-bib-0014] Fazeli, M. , Alizadeh, M. , & Pirsa, S. (2022). Nanocomposite film based on gluten modified with *Heracleum persicum* essence/MgO/Polypyrrole: Investigation of physicochemical and electrical properties. Journal of Polymers and the Environment, 30(3), 954–970.

[fsn34411-bib-0015] Gao, H. X. , He, Z. , Sun, Q. , He, Q. , & Zeng, W. C. (2019). A functional polysaccharide film forming by pectin, chitosan, and tea polyphenols. Carbohydrate Polymers, 215, 1–7. 30981334 10.1016/j.carbpol.2019.03.029

[fsn34411-bib-0016] Hernandez‐Perez, P. , Flores‐Silva, P. C. , Velazquez, G. , Morales‐Sanchez, E. , Rodríguez‐Fernández, O. , Hernández‐Hernández, E. , Mendez‐Montealvo, G. , & Sifuentes‐Nieves, I. (2021). Rheological performance of film‐forming solutions made from plasma‐modified starches with different amylose/amylopectin content. Carbohydrate Polymers, 255, 117349. 33436191 10.1016/j.carbpol.2020.117349

[fsn34411-bib-0017] Kibar, E. A. A. , Gönenç, İ. , & Us, F. (2010). Gelatinization of waxy, normal and high amylose corn starches. GIDA‐Journal of Food, 35(4), 237–244.

[fsn34411-bib-0018] Kit, İ. (2023). Development of anthocyanins‐based colorimetric film under visible light irradiation for the detection of food spoilage (Master's thesis), Middle East Technical University.

[fsn34411-bib-0019] Lozano‐Navarro, J. I. , Díaz‐Zavala, N. P. , Velasco‐Santos, C. , Melo‐Banda, J. A. , Páramo‐García, U. , Paraguay‐Delgado, F. , García‐Alamilla, R. , Martínez‐Hernández, A. L. , & Zapién‐Castillo, S. (2018). Chitosan‐starch films with natural extracts: Physical, chemical, morphological and thermal properties. Materials, 11(1), 120. 29329275 10.3390/ma11010120 PMC5793618

[fsn34411-bib-0020] Ma, R. , Zhan, J. , Lu, H. , Chang, R. , & Tian, Y. (2022). Interactions between recrystallized rice starch and flavor molecules. Food Hydrocolloids, 124, 107271.

[fsn34411-bib-0021] Misic, D. , Tadic, V. , Korzeniowska, M. , Nisavic, J. , Aksentijevic, K. , Kuzmanovic, J. , & Zizovic, I. (2020). Supercritical fluid extraction of celery and parsley fruit‐chemical composition and antibacterial activity. Molecules, 25(14), 3163. 32664342 10.3390/molecules25143163 PMC7397072

[fsn34411-bib-0022] Moftah, R. F. , El‐Geddawy, M. A. , & Hamdy, R. M. (2024). Phenolic compound profiles and bioactive properties of parsley leaves extract and seeds oil. Journal of Food and Dairy Sciences, 15(2), 7–11.

[fsn34411-bib-0023] Nyiew, K. Y. , Kwong, P. J. , & Yow, Y. Y. (2022). An overview of antimicrobial properties of kombucha. Comprehensive Reviews in Food Science and Food Safety, 21(2), 1024–1053. 35075759 10.1111/1541-4337.12892

[fsn34411-bib-0024] Osés, S. M. , Pascual‐Maté, A. , de la Fuente, D. , de Pablo, A. , Fernández‐Muiño, M. A. , & Sancho, M. T. (2016). Comparison of methods to determine antibacterial activity of honeys against *Staphylococcus aureus* . NJAS – Wageningen Journal of Life Sciences, 78, 29–33.

[fsn34411-bib-0025] Petkoska, A. T. , Daniloski, D. , D'Cunha, N. M. , Naumovski, N. , & Broach, A. T. (2021). Edible packaging: Sustainable solutions and novel trends in food packaging. Food Research International, 140, 109981. 33648216 10.1016/j.foodres.2020.109981

[fsn34411-bib-0026] Pinto, J. P. , D'souza, O. J. , Hiremani, V. D. , Dalbanjan, N. P. , Kumar, S. P. , Narasagoudr, S. S. , Masti, S. P. , & Chougale, R. B. (2023). Functional properties of taro starch reinforced polysaccharide based films for active packaging. Food Bioscience, 56, 103340.

[fsn34411-bib-0027] Pirsa, S. , Mahmudi, M. , & Ehsani, A. (2023). Biodegradable film based on cress seed mucilage, modified with lutein, maltodextrin and alumina nanoparticles: Physicochemical properties and lutein controlled release. International Journal of Biological Macromolecules, 224, 1588–1599. 36346259 10.1016/j.ijbiomac.2022.10.244

[fsn34411-bib-0028] Pirsa, S. , & Mohammadi, B. (2021). Conducting/biodegradable chitosan‐polyaniline film; antioxidant, color, solubility and water vapor permeability properties. Main Group Chemistry, 20(2), 133–147.

[fsn34411-bib-0029] Punoševac, M. , Radović, J. , Leković, A. , & Kundaković‐Vasović, T. (2021). A review of botanical characteristics, chemical composition, pharmacological activity and use of parsley. Archives of Pharmacy, 71, 177–196.

[fsn34411-bib-0030] Ruggero, F. , Carretti, E. , Gori, R. , Lotti, T. , & Lubello, C. (2020). Monitoring of degradation of starch‐based biopolymer film under different composting conditions, using TGA, FTIR and SEM analysis. Chemosphere, 246, 125770. 31901665 10.1016/j.chemosphere.2019.125770

[fsn34411-bib-0031] Suriyatem, R. , Auras, R. A. , & Rachtanapun, P. (2018). Improvement of mechanical properties and thermal stability of biodegradable rice starch–based films blended with carboxymethyl chitosan. Industrial Crops and Products, 122, 37–48.

[fsn34411-bib-0032] Tarique, J. , Sapuan, S. M. , & Khalina, A. (2021). Effect of glycerol plasticizer loading on the physical, mechanical, thermal, and barrier properties of arrowroot (*Maranta arundinacea*) starch biopolymers. Scientific Reports, 11(1), 13900. 34230523 10.1038/s41598-021-93094-y PMC8260728

[fsn34411-bib-0033] Vargas, B. K. , Fabricio, M. F. , & Ayub, M. A. Z. (2021). Health effects and probiotic and prebiotic potential of kombucha: A bibliometric and systematic review. Food Bioscience, 44, 101332.

[fsn34411-bib-0034] Villarreal‐Soto, S. A. , Beaufort, S. , Bouajila, J. , Souchard, J. P. , & Taillandier, P. (2018). Understanding kombucha tea fermentation: A review. Journal of Food Science, 83(3), 580–588. 29508944 10.1111/1750-3841.14068

[fsn34411-bib-0035] Wicakso, D. R. , Fortuna, D. , Hernadin, I. A. , Nuryoto, N. , Rumbino, Y. , & Damayanti, A. (2023). Characterization of corn starch edible films by the addition of chitosan as a vegetable oil packaging material. Konversi, 12(2), 62–65.

[fsn34411-bib-0036] Yadav, A. , Kumar, N. , Upadhyay, A. , Pratibha , & Anurag, R. K. (2023). Edible packaging from fruit processing waste: A comprehensive review. Food Reviews International, 39(4), 2075–2106.

[fsn34411-bib-0037] Yorghanlu, R. A. , Hemmati, H. , Pirsa, S. , & Makhani, A. (2022). Production of biodegradable sodium caseinate film containing titanium oxide nanoparticles and grape seed essence and investigation of physicochemical properties. Polymer Bulletin, 79(10), 8217–8240.

